# The validity of the information on date of diagnosis of intracranial tumors in the Swedish cancer register

**DOI:** 10.1007/s10654-025-01308-8

**Published:** 2025-10-25

**Authors:** Magnus Kaijser, Maria Feychting, Stefan Skare, Fang Fang

**Affiliations:** 1 Institute of Environmental Epidemiology, Karolinska Institute, Stockholm, Sweden; 2 Department of Clinical Neuroscience Sciences, Karolinska Institute, Stockholm, Sweden; 3 Department of Neuroradiology, Karolinska University Hospital, Eugeniavägen 3, B5:20, 171 76 Stockholm, Sweden

**Keywords:** Cancer, Cancer register, Date of diagnosis

## Abstract

Purpose: To assess the validity of information on the date of diagnosis of intracranial tumors in the Swedish National Cancer Register. Materials and Methods: The study was conducted in Region Stockholm, covering approximately 2.4 million inhabitants. Data from the Image and Function Service (BFT) archive, which contains a population-based database of all diagnostic radiology in the Stockholm Region, was used. The study included all primary cases of intracranial tumors (ICD10 codes C70 and C71) reported from Stockholm to the National Cancer Register from 2010 to 2020. Radiology reports from CT and MRI exams performed from 2000 to 2020 were reviewed. Reports from exams conducted more than one month before the Swedish Cancer Register diagnosis were manually examined to validate the date of diagnosis. Results: For 98.8% of the brain tumor patients and 96.2% of the meningioma patients, the date of first radiological diagnosis was one month or less prior to date of diagnosis in the Swedish Cancer Register. Of the patients with radiological diagnoses more than one month prior to cancer register diagnosis, 30 patients (1.0%) had tumors reported to the register one month to one year after radiological diagnosis, and 36 patients (1.2%) had tumors reported more than one year later. Conclusion: The study found that for patients from the Stockholm Region with intracranial tumors reported to the Swedish Cancer Register during the period 2010 through 2020, date of diagnosis is highly accurate.

## Introduction

Several studies have shown an increased risk of brain tumor after exposure to ionizing radiation of the head from computed tomography (CT) [1–5]. Unless the brain tumor is reported at autopsy, a radiological examination of the head is virtually a prerequisite to obtain a diagnosis, and CT is the most commonly used tool in radiology of the head. Thus, the results from these studies may in some part be biased by reversed causality, i.e. the tumor led to a CT examination rather than the CT examination being the cause of the tumor. A suggestion of this was presented in a cohort study by Nordenskjold and colleagues on the risk of meningioma among patients examined by CT of the head in the 1970s and 1980s [6]. In this study, a crude analysis showed that the risk of meningioma was higher among exposed subjects compared to population controls. Since the researchers had access to the radiology reports from the exams that constituted the exposure, the meningioma cases could be validated. It was found that some of the tumor patients had been subjected to other radiation treatment to the head and, most notably, some of the tumor cases had been reported to the National Cancer Register as incident during the follow-up period albeit being present already at the initial CT exam. When these cases were excluded, there was no longer any significantly increased risk of meningioma among those exposed to CT of the head. This study was small, however, but highlights delayed reporting of radiologically detected tumors to the National cancer register as a potentially important source of bias in cohort studies of brain tumors after CT exposure of the head. Delayed reporting may lead to the inclusion of prevalent cases into the study, that are often followed in clinical routine by repeated radiological examinations to the head. Thus, they will appear to being exposed to higher doses of ionizing radiation.

The aim of this study was to validate the date of diagnosis for intracranial tumors registered in the National Cancer Register and to assess the extent of delayed reporting of radiologically detected intracranial tumors, through the use of a new population-based database of all diagnostic radiology performed in the Region Stockholm.

## Methods

### Setting

The study was conducted in the county of Stockholm (Region Stockholm), comprising approximately 2,4 million inhabitants. In Region Stockholm, there is a shared radiology archive called the Image and Function Service (Bild- och Funktionstjänsten, BFT), which contains information from all radiology departments that have agreements with the region. As all the private radiology services in Stockholm also have agreements with Region Stockholm, this means that BFT has a virtually complete population-based archive of all radiological examinations in the region.

The coverage of radiological examinations backward in time varies and depends on when the different radiology departments were digitized, something that occurred around the turn of the millennium. There is also variation in coverage for different types of data, where the written radiology reports have the best coverage and are saved for the entire BFT from when digitization started in the early 2000s and onwards, while the referral notes and the actual images have later dates for complete coverage. From 2009 onwards, digitization was complete, and both referral notes containing medical history and queries, radiological reports, and images are saved for all examinations in the archive.

### Study participants and methods

The study included all patients with a diagnosis of a primary intracranially located tumor of the meninges and brain parenchyma, according to ICD-10 codes C70 (meningiomas) and C71 (brain tumors) that were reported to the Regional Cancer Centre in Stockholm, the entity to which health care providers in Region Stockholm report to the Swedish National Cancer Register (“cancer register”). We included all incident cases during the period 2010 through 2020. For these patients, all reports from examinations of the head with computed tomography (CT) and magnetic resonance imaging (MRI) were collected from the BFT for the period 2000 through 2020. All reports from head scans performed more than one month prior to date of diagnosis were examined manually to assess diagnoses of intracranial tumors, i.e. intraparenchymal brain tumors and meningiomas. The month of first occurrence of an intracranial tumor in the radiology report was then compared to the month of diagnosis in the Cancer Register.

Since the study is descriptive, we abstained from statistical testing and only number of cases and proportions tabulated by sex and time between radiological and register diagnosis are presented.

## Results

There were 1096 patients with meningioma and 2012 patients with intraparenchymal brain tumors reported to the Cancer Register during the period from 2010 to 2020, and these patients had a total of 36 926 MRI and CT scans of the brain performed from 2000 to 2020. In 68.1% of the meningioma patients, the first scan of the head was a CT scan, and the corresponding figure for brain tumor patients was 76.2%. There were 21 patients with brain tumor and 14 patients with meningioma that had no radiological information. Of all the scans, 5.3% were done more than one month prior to the date of diagnosis recorded in the Cancer Register (i.e., register diagnosis). The total number of scans in the cohort are reported in further detail in Table 1.

**Table 1:** Number of CT and MRI scans of the head from 2000 through 2020 prior to date of diagnosis registered in the National cancer register among patients with primary brain tumor or meningioma diagnosed during 2010–2020, by tumor type and sex

Time prior to date of diagnosis in the cancer register	Number of scans								
	All tumor patients			Brain tumor patients			Meningioma patients		
	All	Men	Women	All	Men	Women	All	Men	Women
Total	36,926	17,815	19,111	24,963	14,141	10,822	11,963	3674	8289
≤ 1 month	34,961	16,902	18,059	23,725	13,506	10,219	11,236	3396	7840
1 year	354	174	180	281	152	129	73	22	51
2 years	174	67	104	123	52	71	48	15	33
3 years	137	64	73	96	51	45	41	13	28
4 years	150	63	87	105	45	60	45	18	27
≥ 5 years	1153	545	608	633	335	298	520	210	310

When assessing the time of first scan for each individual in the cohort, regardless of whether it showed a tumor or not, 24.2% of the patients had a brain scan at one month or more preceding the register diagnosis, and 15.9% had a scan four years or more before the register diagnosis (Table 2).

**Table 2:** Time between first CT or MRI scan of the head and cancer register diagnosis among patients with a primary brain tumor or meningioma diagnosed during 2010–2020, by tumor type and sex

Time prior to diagnosis			Time from first scan to register diagnosis												
	All patients		Brain tumor patients							Meningioma patients					
			All		Men		Women			All		Men		Women	
	n	%	n	%	n	%	n	%		n	%	n	%	n	%
Total	3108	100	2012	100	1161	100	851	100		1096	100	322	100	774	100
≤ 1month	2357	75,8	1510	75,0	875	75,4	635	74,6		847	77,3	256	79,5	591	76,4
> 1 month - <1 year	112	3,6	88	4,4	53	4,6	35	4,1		24	2,2	7	2,2	17	2,2
1 - <2 years	46	1,5	35	1,7	17	1,5	18	2,1		11	1,0	2	0,6	9	1,2
2 - <3 years	47	1,5	36	1,8	21	1,8	15	1,8		11	1,0	2	0,6	9	1,2
3 - <4 years	53	1,7	34	1,7	20	1,7	14	1,6		19	1,7	4	1,2	15	1,9
≥ 4 years	493	15,9	309	15,4	175	15,1	134	15,7		184	16,8	51	15,8	133	17,2

When reading the reports of the 1968 scans performed one month or more prior to the register diagnosis, we found only 24 patients with a radiologically detected brain tumor and 42 with a radiologically detected meningioma that were reported to the Cancer Register more than one month after radiological diagnosis. Thus, for 98.8% of the brain tumor patients and 96.2% of the meningioma patients, the date of first radiological diagnosis was one month or less prior to date of diagnosis in the Swedish Cancer Register. The time between radiological diagnosis and the reporting of primary brain tumors and meningiomas to the Cancer Register is described in further detail in Table 3.

**Table 3:** Time from radiological diagnosis of primary brain tumor and meningioma to date of diagnosis registered in the Swedish cancer register, among patients diagnosed between 2010–2020, by tumor type and sex*

Time prior to diagnosis			Time from radiological diagnosis to cancer register diagnosis											
	All patients		Brain tumor						Meningioma					
			All		Men		Women		All		Men		Women	
	n	%	n	%	n	%	n	%	n	%	n	%	n	%
Total	3108	100	2012	100	1161	100	851	100	1096	100	322	100	774	100
≤ 1month	3042	97,9	1988	98,8	1152	99,2	836	98,2	1054	96,2	312	96,9	742	95,9
> 1 month - <1 year	30	1,0	15	0,7	5	0,4	10	1,2	15	1,4	4	1,2	11	1,4
1 - <2 years	9	0,3	3	0,1	2	0,2	1	0,1	6	0,5	1	0,3	5	0,6
2 - <3 years	4	0,1	1	0,0	1	0,1	0	0,0	3	0,3	0	0,0	3	0,4
3 - <4 years	3	0,1	0	0,0	0	0,0	0	0,0	3	0,3	1	0,3	2	0,3
≥ 4 years	20	0,6	5	0,2	1	0,1	4	0,5	15	1,4	4	1,2	11	1,4

* = Based on all scans of the head performed during the period 2000–2020

Among meningioma patients, there were seven patients aged 0–17 years with a register diagnosis and none of these had a radiological diagnosis that preceded register diagnosis with more than one month. For the 199 brain tumors that were diagnosed among children, however, the corresponding number of patients was 13. Thus, more than half of the 24 cases where radiological and register diagnoses differed with more than one month occurred among children (Table 4).

**Table 4:** Time from radiological diagnosis of primary brain tumor to date of diagnosis registered in the Swedish cancer register, among patients diagnosed between 2010–2020, by age*

Time prior to diagnosis	Time from radiological diagnosis to cancer register diagnosis					
	Brain tumor					
	All		0–17 years		18 + Years	
	n	%	n	%	n	%
Total	2012	100	199	100	1813	100
≤ 1month	1988	98,8	186	93,5	1802	99,4
> 1 month - <1 year	15	0,7	12	6,0	3	0,2
1 - <2 years	3	0,1	0	0,0	3	0,2
2 - <3 years	1	0,0	1	0,5	0	0,0
3 - <4 years	0	0,0	0	0,0	0	0,0
≥ 4 years	5	0,2	0	0,0	5	0,3

* = Based on all scans of the head performed during the period 2000–2020

## Discussion

In this study using a population-based archive of radiology reports of brain scans from up to 20 years prior to the date of diagnosis of brain tumors and meningiomas as recorded in the Swedish Cancer Register, we found that for 97.9% of the patients, register date of diagnosis corresponded within a month to first radiological diagnosis. This suggests that delayed reporting of brain tumors to the Cancer Register is less of a problem in the 2010s than what it may have been in studies from previous decades [6]. In the present study, 97.5% of the patients with meningiomas had a date of diagnosis registered in the Cancer Register within one year of first radiological diagnosis, and for parenchymal brain tumors, the corresponding figure was 99.5%.

The strength with this study is that it is based on a population-based archive of radiological records. Since a radiological diagnosis is a prerequisite for any subsequent steps in the treatment chain of intracranial tumors, the data in our study, excluding autopsies, represents the initial source from which all reporting of such tumors to the Cancer Register and other health registers is derived. Reporting of intracranial tumors has been found to vary between health registers, and patients reported to one register are not necessarily reported to the others [7–9]. It can therefore be argued that the kind of data that forms the basis of our study are the most complete regarding detection of intracranial tumors. Apart from this apparent strength, our study has some limitations. First and foremost, our study has no coverage of radiologically detected tumors that are never reported to the Swedish Cancer Register. In a study during 1990–2014, it was found that 23% of brain tumors reported to the Swedish Inpatient Register were never reported to the Swedish Cancer Register, and for benign tumors, the proportion was as high as 36% [9]. While the specificity of diagnosis in the Inpatient Register remains to be assessed, underreporting of intracranial tumors to the Swedish Cancer Register might also be an issue. Although this limitation makes it impossible to deduce from our study the completeness of the Cancer Register overall in the coverage of intracranial tumors, it does not, however, threaten the validity of our results regarding the accuracy of the date of diagnosis for the cases that are indeed reported. Another limitation is that we had no information on when the tumors were reported to the Cancer Register. This makes it possible that intracranial tumors, when reported to the Cancer Register, are assigned as date of diagnosis the date when the cancer was first identified on a radiological exam, even though the reporting occurred years later. Another limitation is that our study encompasses only a limited period of the entire time period of the Swedish Cancer Register, which was initiated in 1958. Although this limitation reduces the possibility to extrapolate our results to the period prior to 2010, our results are relevant for contemporary use. A fourth limitation is that we included only data from Region Stockholm in the study instead of the entire population of Sweden. Although the regulation for reporting to the Swedish Cancer Register is the same all over the nation, there may be differences in access to radiology and in how clinicians handle radiology reports of incident intracranial tumors. Caution may therefore be warranted when extrapolating our data to the entire Swedish Cancer Register.

This study is, to our knowledge, the first study that validates date of diagnosis for intracranial tumors registered in the Swedish Cancer Register, and it is the first study that uses a population-based radiological archive for the validation. We found a very low percentage of brain and meningeal tumors to have been detected on CT and MRI scans of the brain more than one month prior to the registered date of diagnosis in the Cancer Register. Our study differs from the results of the study of meningioma by Nordenskjold et al. where more than 20% of the patients reported to the Swedish Cancer Register had a meningioma or a brain tumor already at the first CT [6]. The explanation of this contrast is most likely the changed regulations of reporting to the Cancer Register, and this partly demonstrates that our results must be extrapolated with caution to the period before 2010.

The type of radiological data used in this study holds great promise for further research. Diagnostic radiology plays a pivotal role in modern healthcare, and with the advent of new machine learning techniques, it is now possible to categorize much larger datasets of radiological reports than was previously feasible. This enables more detailed studies of both cancer and other diseases, with a level of granularity that could not be achieved using register data alone.

In conclusion, we found that, in the Swedish Cancer Register during 2010–2020, the information on date of diagnosis for intracranial tumors is highly accurate, with only 1.2% of brain tumor patients and 3.8% of meningioma patients having a radiological tumor diagnosis that precedes the register diagnosis with more than one month.
